# Energy metabolism of *Heliobacterium modesticaldum *during phototrophic and chemotrophic growth

**DOI:** 10.1186/1471-2180-10-150

**Published:** 2010-05-24

**Authors:** Kuo-Hsiang Tang, Hai Yue, Robert E Blankenship

**Affiliations:** 1 Department of Biology, Campus Box 1137, Washington University in St. Louis, One Brookings Drive, St. Louis, Missouri 63130, USA; 2 Department of Chemistry, Campus Box 1137, Washington University in St. Louis, One Brookings Drive, St. Louis, Missouri 63130, USA

## Abstract

**Background:**

*Heliobacterium modesticaldum *is a gram-positive nitrogen-fixing phototrophic bacterium that can grow either photoheterotrophically or chemotrophically but not photoautotrophically. Surprisingly, this organism is lacking only one gene for the complete reverse tricarboxylic acid (rTCA) cycle required for autotrophic carbon fixation. Along with the genomic information reported recently, we use multiple experimental approaches in this report to address questions regarding energy metabolic pathways in darkness, CO_2 _fixation, sugar assimilation and acetate metabolism.

**Results:**

We present the first experimental evidence that D-ribose, D-fructose and D-glucose can be photoassimilated by *H. modesticaldum *as sole carbon sources in newly developed defined growth medium. Also, we confirm two non-autotrophic CO_2_-fixation pathways utilized by *H. modesticaldum*: reactions catalyzed by pyruvate:ferredoxin oxidoreductase and phosphoenolpyruvate carboxykinase, and report acetate excretion during phototrophic and chemotrophic growth. Further, genes responsible for pyruvate fermentation, which provides reducing power for nitrogen assimilation, carbon metabolism and hydrogen production, are either active or up-regulated during chemotrophic growth. The discovery of ferredoxin-NADP^+ ^oxidoreductase (FNR) activity in cell extracts provides the reducing power required for carbon and nitrogen metabolisms. Moreover, we show that photosynthetic pigments are produced by *H. modesticaldum *during the chemotrophic growth, and demonstrate that *H. modesticaldum *performs nitrogen fixation during both phototrophic and chemotrophic growth.

**Conclusion:**

Collectively, this report represents the first comprehensive studies for energy metabolism in heliobacteria, which have the simplest known photosynthetic machinery among the entire photosynthetic organisms. Additionally, our studies provide new and essential insights, as well as broaden current knowledge, on the energy metabolism of the thermophilic phototrophic bacterium *H. modesticaldum *during phototrophic and chemotrophic growth.

## Background

Several features characterize the physiological and metabolic aspects of phototrophic heliobacteria [1-5]: (a) They are the only known phototrophs that belong to the gram-positive bacterial phylum *Firmicutes*, and as is typical of members of this group, which includes species of *Bacillus *and *Clostridium*, heliobacteria can form heat resistant endospores; (b) They produce the unique pigment bacteriochlorophyll *g *(BChl *g*); (c) They produce 8^1^-hydroxy-chlorophyll *a *with a farnesol tail (8^1^-OH-Chl *a*_F_), which serves as the primary electron acceptor from the reaction center (RC) special pair; (d) They contain a type I homodimeric RC bound to the cytoplasmic membrane; (e) They require organic carbon sources for both phototrophic growth and chemotrophic (fermentative) growth; and (f) they are active nitrogen-fixers and also produce hydrogen. Further, *Heliobacterium modesticaldum*, isolated from hot springs microbial mats and volcanic soils in Iceland [6], is one of only two known anoxygenic phototrophs that can fix nitrogen at temperatures higher than 50°C (the other is *Chlorobaculum tepidum*) [7,8].

Genomic sequence data of *H. modesticaldum *suggests that several genes required for the known autotrophic carbon fixation pathways are missing [1]. This is consistent with previous physiological studies indicating that heliobacteriaceae are obligate heterotrophs [2]. In the absence of known CO_2_-fixation mechanisms, it is unknown whether alternative pathways may be adapted by *H. modesticaldum *for CO_2 _assimilation. The genomic information suggests that one candidate for anaplerotic CO_2 _incorporation is phosphoenolpyruvate (PEP) carboxykinase. We recently identified the non-autotrophic, anaplerotic CO_2 _assimilation mechanism in the photoheterotrophic α-proteobacterium *Roseobacter denitrificans *[9]. Whether a similar anaplerotic pathway and/or other pathways are employed for CO_2 _incorporation in *H. modesticaldum *has not been verified.

It has been reported that pyruvate, lactate, acetate, and yeast extract can support photoheterotrophic growth of *H. modesticaldum *[2,6]. Although essential genes in the oxidative pentose phosphate (PP) and Entner-Doudoroff (ED) pathways are absent in the genome, genes for the Embden-Meyerhof-Parnas (EMP) pathway (glycolysis), gluconeogenesis, and a ribose ATP-binding cassette (ABC) transporter (*rbsABCD*) have been annotated in the genome. However, neither hexose nor ribose has been reported to support the growth of *H. modesticaldum *[3]. Additionally, while the most vigorous growth of *H. modesticaldum *occurs photoheterotrophically, *H. modesticaldum *can also grow chemotrophically (dark, anoxic) by fermentation [6]. But heliobacterial energy metabolism during chemotrophic (fermentative) growth is not fully understood. To address these questions about the carbon and energy metabolism of *H. modesticaldum*, experimental evidence gathered using a multi-faceted approach and working hypotheses are presented in this report.

## Results

### D-ribose, D-fructose and D-glucose can support the growth of *H. modesticaldum*

**Figure 1:**
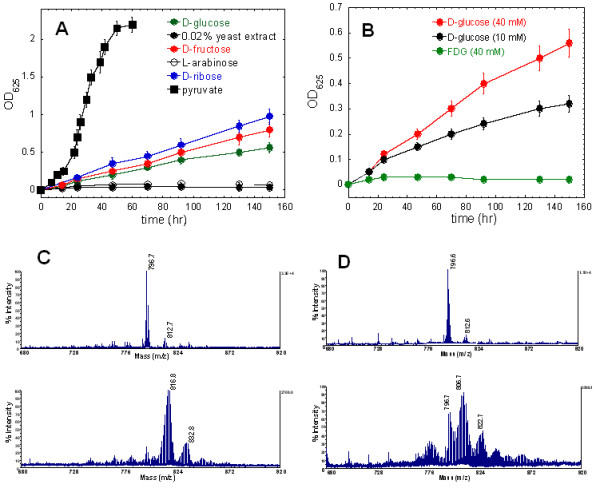
**Phototrophic growth of *H. modesticaldum *on pyruvate and various sugars, and mass spectra of (bacterio)chlorophylls extracted from cells grown on pyruvate and glucose**. Growth of *H. modesticaldum *on 20 mM pyruvate, 40 mM sugars, or 0.02% yeast extract (A), and on 10 mM D-glucose, 40 mM D-glucose, or 40 mM 2'-fluoro-2'-deoxy-D-glucose (FDG) (B) as defined carbon source in the growth medium. Either no or only "vitamin-level" (0.02%) yeast extract is included in the growth medium, and detailed growth conditions are described in Materials and Methods. Mass spectra of (bacterio)chlorophylls extracted from cultures grown on pyruvate (I, upper panel) vs. [3-^13^C]pyruvate (II, lower panel) in PMS medium (C) and glucose (I, upper panel) vs. [U-^13^C_6_]glucose (II, lower panel) in YE medium (D).

Only a few defined carbon sources, lactate, acetate (in the presence of HCO_3_^-^) and pyruvate, and yeast extract, an undefined carbon source, have been reported to support growth of *H. modesticaldum *[2,6]. In order to enhance our understanding of the energy and carbon metabolism of *H. modesticaldum*, it is useful to explore other organic carbon sources. Glucose or fructose are reported to support the growth of *Heliobacterium gestii *but not *H. modesticaldum *[2], whereas a complete EMP pathway has been annotated in the genome of *H. modesticaldum *[1]. In the yeast extract (YE) growth medium with 0.4% yeast extract included, significant cell growth can be detected with 40 mM D-glucose or D-fructose supplied, and cell growth is glucose concentration -dependent (Additional file 1: Figure S1). Although interpretations of these experimental results are complicated by the fact that 0.4% yeast extract alone can support the growth of *H. modesticaldum *[2], photo-assimilation of glucose to generate cell material is identified by mass spectrometry (Figure 1). The mass spectral studies are further elaborated below.

By optimizing the growth conditions, we successfully grew the cultures on D-ribose, D-glucose and D-fructose in the growth medium containing 0.02% yeast extract (i.e. "vitamin level" yeast extract), whereas no growth can be detected with only 0.02% yeast extract in the culture medium (Figure 1A). Cell growth is dependent on the concentration of D-sugars, and no growth of *H. modesticaldum *is seen with 40 mM 2'-fluoro-2'-deoxy-D-glucose (FDG) as the sole carbon source (Figure 1B). Lack of the growth on FDG, a glucose analogue, is consistent with the mechanism of action of FDG that cannot be metabolized inside the cells because it lacks the 2'-hydroxyl group in normal glucose required for conversion of D-glucose-6-phosphate to D-fructose-6-phosphate in glycolysis. Alternatively, no growth is detected on L-arabinose, which is one of the most abundant pentoses present as a constituent of bacterial cell wall and is a more common isomer than D-arabinose. Many bacteria contain an inducible operon that encodes a series of enzymes and transporters that allows L-arabinose to be used as a sole carbon source in cell culture. No arabinose transporter (*araE*) is annotated in the genome of *H. modesticaldum*.

In addition to physiological studies, we also determine the uptake of D-hexose and assay the enzymatic activity for the enzymes specific for the EMP pathway. Our studies indicate 20-25% D-fructose (8-10 mM) and ~10% D-glucose (~4 mM) being assimilated, consistent with better growth on D-fructose than on D-glucose. No acetate is excreted from 40 mM glucose-grown cultures (data not shown). Enzymatic activity of hexokinase (10 nmole/min•mg protein), 6-phosphofructokinase (20 nmole/min•mg protein) and pyruvate kinase (10 nmole/min•mg protein), three enzymes specific for the EMP pathway and not shared with the gluconeogenesis pathway, can be detected in hexose-grown cultures. Together, our studies indicate that *H. modesticaldum *uses the EMP pathway for carbohydrate metabolism when D-glucose, D-fructose and D-ribose are supplied as carbon sources.

### Probe glucose photoassimilation by mass spectrometry

As described above, glucose and fructose can enhance the growth of *H. modesticaldum *in YE medium (Additional file 1: Figure S1). We have investigated the roles of glucose in the cultures grown on glucose and 0.4% yeast extract. In addition to the experimental evidence presented above, we determined the molecular mass of photosynthetic pigments of *H. modesticaldum*, BChl *g *and 8^1^-OH-Chl *a*_F _(BChl *g*, 819 Da; 8^1^-OH-Chl *a*_F_, 835 Da), in glucose-grown cultures by MALDI-TOF mass spectrometry. If glucose is photo-assimilated via the EMP pathway to produce cell materials of *H. modesticaldum*, BChl *g *and 8^1^-OH-Chl *a*_F _should be labeled when ^13^C-labeled glucose (Glc) is added to the growth medium. To test the hypothesis, we obtain mass spectra of (B)Chls extracted from pyruvate-grown cultures as the positive control, since pyruvate has been established as the sole carbon source for *H. modesticaldum*. (B)Chls were extracted as reported previously [10]. Because an acidic matrix (*α*-cyano-4-hydroxycinnamic acid) was used to prepare the samples submitted to mass spectral analysis, peaks corresponding to demetallization of BChl *g *and 8^1^-OH-Chl *a*_F _were detected (upon demetallization (M-22; - Mg^2+ ^+ 2 H^+^): BChl *g*, 797 Da; 8^1^-OH-Chl *a*_F_, 813 Da) (Figure 1C, upper panel, and Figure 1D, upper panel). Compared to the sample from unlabeled pyruvate-grown cultures (Figure 1C, upper panel), higher molecular masses corresponding to labeled (B)Chls (BChl *g*, 817 Da; 8^1^-OH-Chl *a*_F_, 833 Da) were detected using [3-^13^C]pyruvate as the sole carbon source (Figure 1C, lower panel).

**Figure 2:**
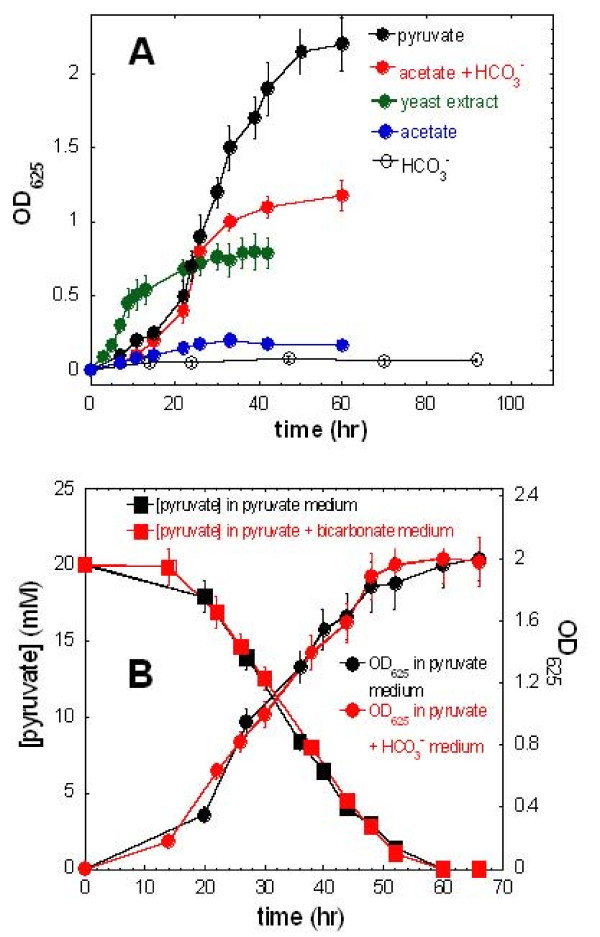
**Growth of *H. modesticaldum *on various carbon sources**. Cell growth on various carbon sources (A), and growth curve versus pyruvate consumption in pyruvate-grown cultures with and without bicarbonate included (B). Other than growth on yeast extract (green curve), the studies presented in Figure 2 were performed in the minimal growth medium without defined carbon source.

Similarly, we determined the molecular mass of (B)Chls from the cultures grown on unlabeled Glc (Figure 1D, upper panel) or [U-^13^C_6_]Glc (Figure 1D, lower panel) in YE medium. Because 0.4% yeast extract alone can support the growth of *H. modesticaldum *(Figure 2A) and produce (B)Chls, unlabeled (B)Chls were detected in the mass spectrum from cell cultures grown in YE medium containing [U-^13^C_6_]Glc (Figure 1D, lower panel). In contrast, less unlabeled BChl *g *was detected in the samples from cultures grown on [3-^13^C]pyruvate as sole carbon source (Figure 1C, lower panel). Nevertheless, The lower panel of Figure 1D shows that most of BChl *g *and 8^1^-OH-Chl *a*_F _molecules are ^13^C-labeled in the samples from [U-^13^C_6_]Glc-grown cultures, since the peaks corresponding to ^13^C-labeled molecular mass of (B)Chls (BChl *g*, 807 Da; 8^1^-OH-Chl *a*_F_, 823 Da, as well as high molecular mass peaks) cannot be detected in unlabeled glucose-grown sample (Figure 1D, upper panel). Together, our studies demonstrate that glucose is transported into cells and photoassimilated to produce cell materials.

### Growth on yeast extract

As previously reported [2], we also found that yeast extract (0.4%) alone can support growth of *H. modesticaldum *(Figure 2A). It is known that many undefined carbon sources, vitamin mixtures and amino acids included, are included in yeast extract. We successfully replaced yeast extract with vitamin B_12 _for supporting the growth of a different photoheterotrophic bacterium [9]. In all of the growth media of *H. modesticaldum*, vitamin B_12 _has always been included, and it is not yet known what carbon sources in the yeast extract support the photoheterotrophic growth of *H. modesticaldum*. With approaches listed in Materials and Methods, we have estimated that the amount of pyruvate, acetate and lactate in yeast extract is negligible. However, the inclusion of pyruvate or acetate as a defined organic carbon source, along with yeast extract, can significantly enhance growth (Additional file 2: Figure S2). Alternatively, it is possible that some amino acids in yeast extract may support the growth of *H. modesticaldum*, and the oxidation of amino acids transported into the cell can generate reducing power and chemical energy. To test this hypothesis, we grew *H. modesticaldum *on casamino acids that contain predominately a mixture of free amino acids, and observed comparable cell growth with 1.0% casamino acids versus with 0.4% yeast extract after 48 hours of growth (OD_625 _is ~0.7-0.8). Also, we didn't observe significant growth enhancement with vitamin mixtures included in casamino acids-grown cultures. Together, our studies support the idea that amino acids contribute to the growth of *H. modesticaldum*. Further, we have probed the contribution of glutamate and glutamine for cell growth of *H. modesticaldum*. Glutamate can serve as a nitrogen source for *H. modesticaldum *[6], while our current studies indicate that either glutamate or glutamine (up to 100 mM each) cannot support the growth of *H. modesticaldum *as a sole carbon source during phototrophic and chemotrophic growth.

To investigate the impact of yeast extract on metabolic pathways, we compared transcriptomic data from cultures containing PYE (pyruvate and yeast extract are carbon sources) and PMS (pyruvate as the sole organic carbon) growth media (all of the growth media are described in Materials and Methods section and Table 1). It is generally assumed that proteomic and transcriptomic data are related [11], and that higher mRNA levels normally lead to more protein production, particularly in prokaryotes with no mechanism of post-transcriptional modification. Our data show that the addition of yeast extract to the culture media has little effect on the transcriptional levels of most genes involved in carbon metabolism and other cellular functions (Additional file 3: Table S1).

**Table 1:** Organic carbon sources used in growth media reported in this paper.

abbreviation	Growth medium	Carbon sources	Reference
PMS	pyruvate-mineral salt medium	pyruvate (20 mM)	[2]
PYE	pyruvate-yeast extract medium	pyruvate (phototrophic growth, 20 mM; chemotrophic growth, 40 mM), yeast extract (0.4%)	[2]
YE	yeast extract medium	yeast extract (0.4%)	[2]
AMS	acetate-mineral salt medium	acetate (40 mM), HCO_3_^- ^(20 mM)	[2] and this report
	hexose- and ribose-grown medium	sugar (hexose or ribose, 40 mM), yeast extract (0.02%)	[2] and this report

### Non-autotrophic CO_2 _assimilation by *H. modesticaldum*

It has been recognized that pyruvate is the preferred organic carbon source for heliobacteria and it can support both photoheterotrophic and chemotrophic growth [3]. Consistent with previous reports, our studies show that *H. modesticaldum *grows better using pyruvate as carbon source compared to other organic carbon sources (Figure 2A), and the rate of cell growth corresponds to that of pyruvate consumption (Figure 2B). In contrast to CO_2_-enhanced growth of *Chlorobaculum *(*Cba*.) *tepidum *and other green sulfur bacteria [12], no difference in growth rate can be detected with or without 0.4% HCO_3_^- ^included in pyruvate-grown cultures (Figure 2B). Moreover, no growth can be detected with HCO_3_^- ^as the sole carbon source (Figure 2A).

The lack of autotrophic growth in *H. modesticaldum *can be attributed to the lack of a gene encoding ATP citrate lyase (ACL) [1,5], which catalyzes the cleavage of citrate to acetyl-CoA and oxaloacetate (OAA) and is one of the key enzymes specific in the autotrophic CO_2 _fixation via the reductive (or reverse) tricarboxylic acid (rTCA) cycle [13-15]. To confirm the absence of an enzyme having ACL activity, we performed activity assays in cell-free extracts of *H. modesticaldum *and *Cba. tepidum*. The latter served as a positive control for ACL activity, which is documented in *Cba. tepidum *[16,17]. Consistent with previous reports, the activity of ACL was clearly detected in cell free extracts of *Cba. tepidum*, but not in *H. modesticaldum *(Additional file 4: Figure S3). Additionally, the activity of citrate synthase, catalyzing the formation of citrate from condensation of OAA and acetyl-CoA in the oxidative TCA cycle, also cannot be detected (data not shown).

Alternatively, the genomic data suggest that certain non-autotrophic pathways may be available for CO_2 _assimilation in *H. modesticaldum *[1]. The *pckA *gene (HM1_2773), encoding phosphoenolpyruvate (PEP) carboxykinase (PEPCK), has been annotated in the genome of *H. modesticaldum*. The activity of PEPCK (30 nmole/min•mg protein) was detected in cell-free extracts of *H. modesticaldum *and *pckA *is expressed, based on QRT-PCR analysis, in all of the growth conditions tested (Table 2 and Additional file 3: Table S1). Together, our experimental data indicate that *H. modesticaldum *uses PEPCK to assimilate CO_2 _and generates ATP via substrate-level phosphorylation (PEP + ADP + CO_2 _→ OAA + ATP), in agreement with previously proposed carbon metabolic pathways in heliobacteria [1,18].

**Table 2:** Gene expression profiles for phototrophic (light) vs. chemotrophic (dark) growth.

Gene name	ΔC_T_*^a ^*(light)	ΔC_T_*^a ^*(dark)	ΔΔC_T_*^b^*	Relative gene expression level (light/dark)*^c^*
**Genes for carbon metabolism**				

*pfkA *(6-phosphofructokinase)	15.0 ± 0.1	22.0 ± 0.1	7.0 ± 0.2	128
*pykA *(pyruvate kinase)	13.5 ± 0.1	19.5 ± 0.1	6.0 ± 0.2	64
*porA *(pyruvate:Fd oxidoreductase)	13.7 ± 0.1	11.6 ± 0.0	-2.1 ± 0.1	0.2
*fdxR *(Fd-NADP^+ ^oxidoreductase)	14.7 ± 0.1	15.2 ± 0.1	0.5 ± 0.2	1.4
Ferredoxin	13.4 ± 0.1	13.2 ± 0.1	-0.2 ± 0.2	1
*pshB *(ferredoxin)	14.0 ± 0.1	14.3 ± 0.1	0.3 ± 0.2	1
*ackA *(acetate kinase)	10.6 ± 0.1	12.2 ± 0.1	1.6 ± 0.2	3
*acsA *(acetyl-CoA synthase)	15.5 ± 0.1	21.0 ± 0.1	5.5 ± 0.2	45
*ppdK *(pyruvate phosphate dikinase)	13.4 ± 0.1	17.4 ± 0.1	4.0 ± 0.2	16
*pckA *(PEP carboxykinase)	14.1 ± 0.1	17.2 ± 0.1	3.1 ± 0.2	8
*mdh *(malate dehydrogenase)	14.5 ± 0.1	14.6 ± 0.1	0.1 ± 0.2	1

**Genes for pigment biosynthesis**				

*bchY*	13.1 ± 0.1	15.7 ± 0.0	2.6 ± 0.1	6
*bchB*	14.0 ± 0.0	18.0 ± 0.1	4.0 ± 0.1	16
*bchE*	13.2 ± 0.1	15.0 ± 0.1	1.8 ± 0.2	4
*bchG*	12.9 ± 0.1	13.9 ± 0.1	1.0 ± 0.2	2

**Genes for nitrogen assimilation and hydrogen production**				

*nifK *(Fe/Mo nitrogenase, β subunit)	13.0 ± 0.0	21.5 ± 0.1	8.5 ± 0.1	365
*nifD *(Fe/Mo nitrogenase, α subunit)	13.7 ± 0.0	21.4 ± 0.1	7.7 ± 0.1	197
*hupS *([NiFe]-hydrogenase small subunit)	13.3 ± 0.1	18.4 ± 0.1	5.1 ± 0.2	34
*hupL *([NiFe]-hydrogenase large subunit)	12.7 ± 0.1	18.3 ± 0.1	5.6 ± 0.2	49
*hymD *(Fe only hydrogenase, Hymd subunit)	13.4 ± 0.1	18.7 ± 0.1	5.3 ± 0.2	40
*nuoE*	14.3 ± 0.2	19.7 ± 0.1	5.4 ± 0.3	43
*nuoF*	12.9 ± 0.2	18.6 ± 0.1	5.7 ± 0.3	51
*nuoG*	12.9 ± 0.1	18.6 ± 0.1	5.7 ± 0.2	51

*^a^*ΔC_T _= C_T _(the threshold cycle) of the target gene - C_T _of the 16S rRNA gene [31]

*^b^*ΔΔC_T _= ΔC_T _(dark) - ΔC_T _(light)

*^c ^*relative expression level is = 2^ΔΔ^^C^_T_

### Acetate can serve as a carbon source with CO_2_-enhanced growth

Figure 2A shows that *H. modesticaldum *can be grown with acetate as the sole organic carbon source, and CO_2_-enhanced growth is clearly detected in acetate-grown culture with the addition of exogenous HCO_3_^- ^(0.4%). In contrast, no CO_2_-enhanced growth was detected using pyruvate as the defined organic carbon source (Figure 2B). These studies suggest that pyruvate:ferredoxin oxidoreductase (PFOR) contributes to CO_2_-enhanced phototrophic growth through conversion of acetyl-CoA to pyruvate (equation 1) and is one of the major pathways for CO_2 _assimilation in *H. modesticaldum*.(1)

where Fd_red _and Fd_ox _represent the reduced and oxidized form of ferredoxin (Fd), respectively.

The *porA *gene (HM1_0807), encoding PFOR, has been annotated in *H. modesticaldum*. The enzymatic activity of PFOR has been reported in pyruvate-grown cultures of *Heliobacterium *strain HY-3 [18]. In some relatives of the heliobacteria, such as *Clostridium thermoaceticum *and other clostridia, PFOR is linked to the carbon fixation via the reductive acetyl-CoA pathway (i.e. the Wood-Ljungdahl pathway) [19]. While enzymes that function in this autotrophic carbon fixation pathway are commonly found in both methanogens and acetate-producing clostridia [20], they are not found in the genome of *H. modesticaldum *[1].

### Phototrophic versus chemotrophic growth of *H. modesticaldum*

**Figure 3:**
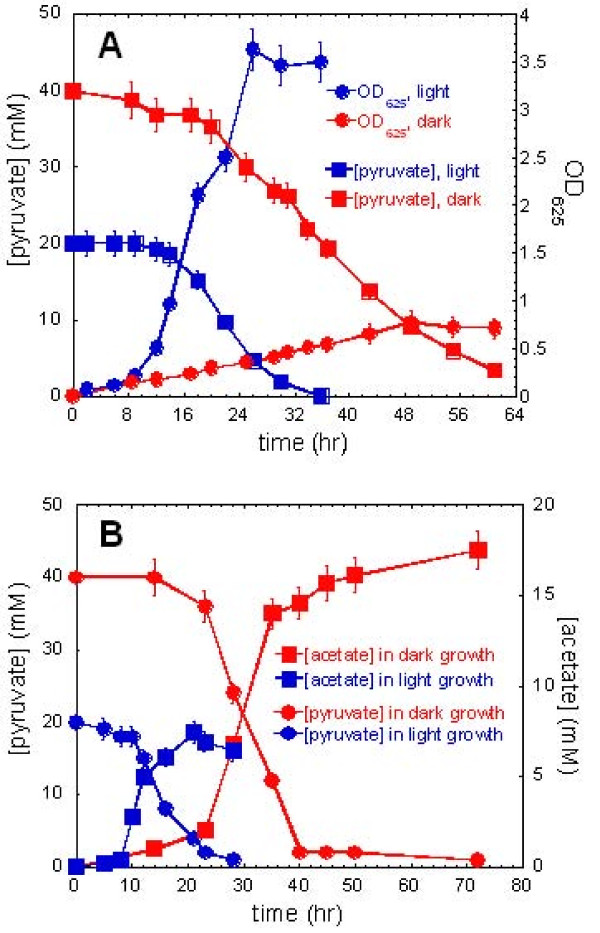
**Cell growth, pyruvate consumption, and acetate production during phototrophic and chemotrophic growth**. 20 mM and 40 mM pyruvate is included in PYE medium during phototrophic and chemotrophic growth, respectively. Cell growth vs. amount of pyruvate (A) and amount of pyruvate and acetate (B) in the cultures during phototrophic growth (blue curve) and chemotrophic growth (red curve) are shown.

*H. modesticaldum *can grow either photoheterotrophically in the light or chemotrophically in the dark [6], but heliobacterial energy metabolism during chemotrophic (fermentative) growth is not well understood. Because pyruvate is a required nutrient for fermentative growth [21] and also best supports phototrophic growth of heliobacteria, the following studies of heliobacterial phototrophic and chemotrophic growth were obtained from cells grown in PYE medium. The OD_625 _of cell cultures and pyruvate consumption during phototrophic and chemotrophic growth are shown in Figure 3A, and the levels of gene expression in each growth condition are reported in Table 2. The major results from our investigation are illustrated below.

### (A) Acetate assimilation and excretion

Figure 3B indicates that acetate is excreted in pyruvate-grown cultures containing 0.4% yeast extract (in PYE medium) during phototrophic and chemotrophic growth, and that the rate of pyruvate consumption generally corresponds to the rate of acetate excretion during chemotrophic and phototrophic growth. Since either pyruvate or acetate can support the phototrophic growth, the amount of acetate production does not increase steadily during phototrophic growth. In contrast, previous reports [2,6] and our studies showed that only pyruvate can support chemotrophic growth of *H. modesticaldum*. When pyruvate is used as the sole carbon source (in PMS medium), the ratio of acetate excretion/pyruvate consumption is similar during phototrophic and chemotrophic growth (35-44%, Table 3). Also, the ratio is comparable in the cultures grown in PYE medium during phototrophic (37%) and chemotrophic growth (40%). Together, these results are coherent with our investigation that no significant amount of pyruvate is included in yeast extract (see "growth on yeast extract"). Additionally, no lactate excretion is detected in pyruvate-grown cultures (Table 3).

**Table 3:** Nutrient uptake and metabolite excretion in PMS medium (pyruvate as the sole carbon source) during various growth conditions.

Growth condition	Nitrogen source	Pyruvate supplied/consumed (mM)	Acetate excretion (mM)	Ratio of pyruvate consumption/acetate excretion	Lactate excretion (mM)
phototrophic growth	NH_4_^+^	20	7.8	39%	--
phototrophic growth + 0.4% bicarbonate	NH_4_^+^	20	7.0	35%	--
phototrophic growth	98% N_2_/ 2% H_2_	20	7.2	36%	--
chemotrophic growth	NH_4_^+^	40	17.5	44%	--
chemotrophic growth	98% N_2_/ 2% H_2_	40	16.8	42%	--

While our results are different from the report of *Heliobacterium *strain HY-3 [18], the authors found more acetate being produced during chemotrophic growth (13.6 mM) than during phototrophic growth (5.9 mM). Both our and their studies demonstrate that acetate can be produced from pyruvate-grown heliobacterial cultures during phototrophic and chemotrophic growth. Two acetate assimilation/excretion pathways are possibly employed by *H. modesticaldum*. One is catalyzed by acetyl-CoA synthetase (ACS, EC 6.2.1.1), proceeding through an acetyl adenylate intermediate; and the other is catalyzed by acetate kinase (ACK, EC 2.7.2.1, acetate ⇌ acetyl-phosphate) and phosphotransacetylase (PTA, EC 2.3.1.8, acetyl-phosphate ⇌ acetyl-CoA) [22]. No ACS activity was reported in the studies of *Heliobacterium *strain HY-3 [18], and it is possible that ACK and PTA are responsible in the acetate assimilation/excretion pathway in *Heliobacterium *strain HY-3.

**Figure 4:**
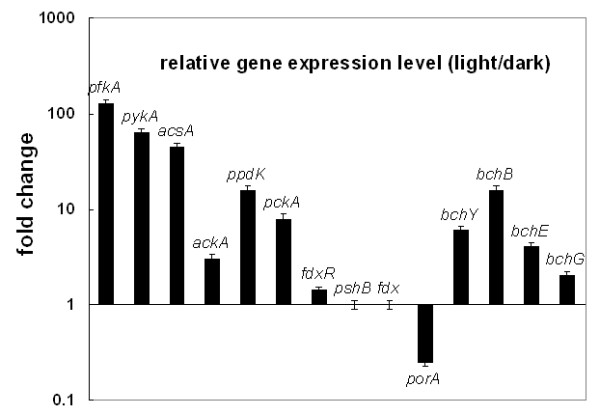
**Relative gene expression levels during phototrophic versus chemotrophic growth**. Only representative genes responsible for carbon metabolism, nitrogen fixation and hydrogen production are shown. Gene name (encoding enzyme): *pfkA *(6-phosphofructokinase), *pykA *(pyruvate kinase), *acsA *(acetyl-CoA synthase), *ackA *(acetate kinase), *ppdK *(pyruvate phosphate dikinase), *pckA *(PEP carboxykinase), *fdxR *(Fd-NADP^+ ^oxidoreductase, FNR), *pshB *(RC core polypeptide, PshB), *fdx *(ferredoxin for FNR), *porA *(pyruvate:Fd oxidoreductase), *bchY *(chlorophyll reductase, subunit Y), *bchB *(protochlorophyllide reductase, subunit B), *bchE *(anaerobic cyclase), and *bchG *(bacteriochlorophyll synthase).

In contrast, genes encoding ACS (*acsA*, HM1_0951) and ACK (*ackA*, HM1_2157), but not PTA (*pta*), have been annotated in the genome of *H. modesticaldum*. The relative gene expression level (the ratio of transcript level in the light/in darkness) of *acsA *is approximately one order of magnitude lower than that of *ackA *(45 versus 3; see Table 2 and Figure 4), whereas the activity of ACS can be only detected in cell extracts of phototrophic growth (Table 4). In contrast, the enzymatic activity of ACK and PTA can be detected in cell extracts of pyruvate-grown cultures during both phototrophic and chemotrophic growth.

**Table 4:** Enzyme activity of enzymes and relative expression level of genes for acetate metabolism in pyruvate-grown cultures during phototrophic and chemotrophic growth.

	Specific activity (nmole/min/mg protein)		
		
Enzyme activity tested	Phototrophic growth	Chemotrophic growth	Relative gene expression level (light/dark)
acetyl-CoA synthetase (ACS)*^a^*	100 ± 20	N/A	45
acetate kinase (ACK)*^a^*	800 ± 40	600 ± 100	3
phosphotransacetylase (PTA)*^a^*	400 ± 50	500 ± 100	--

*^a^*Activity of ACS was determined by a colorimetric assay of PP_i _[39], and activity of ACK and PTA was determined by coupling assays reported previously [18].

**Figure 5:**
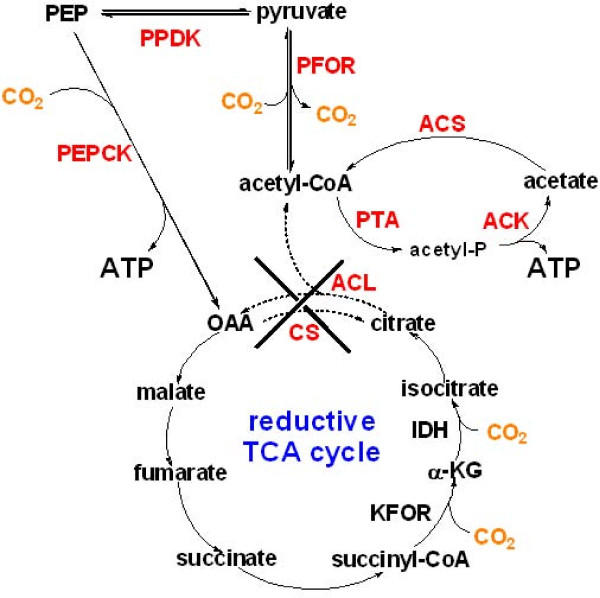
**The proposed carbon flux in *H. modesticaldum***. Abbreviation: ACS, acetyl-CoA synthetase; ACK, acetate kinase; ACL, ATP citrate lyase; CS, citrate synthase; IDH, isocitrate dehydrogenase; α-KG, α-ketoglutarate; KFOR, α-ketoglutarate:ferredoxin oxidoreductase; OAA, oxaloacetate; PEP, phosphoenolpyruvate; PEPCK: phosphoenolpyruvate carboxykinase; PFOR, pyruvate:ferredoxin oxidoreductase; PTA, phosphotransacetylase. Enzymes or pathways investigated in our report are highlighted in red. Dot line represents that the gene is missing and activity is not detected.

Together, our studies suggest that: (i) *H. modesticaldum *uses ACS for catalyzing the conversion of acetate to acetyl-CoA. Lack of enzymatic activity of ACS and low expression of *acsA *in the cultures grown in darkness is consistent with the physiological evidence that acetate cannot support the chemotrophic growth of *H. modesticaldum*; (ii) the gene expression level of *ackA *and enzymatic activity of ACK and PTA are similar during chemotrophic versus phototrophic growth, in agreement with a similar ratio of acetate excretion/pyruvate consumption in light and darkness, indicating that *H. modesticaldum *uses PTA and ACK to convert acetyl-CoA to acetate. ATP is generated via substrate-level phosphorylation in the reaction of acetyl-phosphate being converted to acetate; and (iii) while no *pta *gene has been annotated in the genome, function of PTA is identified in *H. modesticaldum *to convert acetyl-CoA to acetyl-phosphate. Alternatively, some bacteria can use pyruvate oxidase (POX, EC 1.2.3.3, pyruvate + P_i _+ O_2 _⇌ acetyl-phosphate + CO_2 _+ H_2_O_2_) to produce acetyl-phosphate from pyruvate, whereas the O_2_-dependence of POX catalysis is not feasible in the strictly anaerobic bacterium *H. modesticaldum*. Also, no *pox *gene is annotated in the genome. The proposed acetate metabolism of *H. modesticaldum *is shown in Figure 5.

### (B) Gene expression in carbon, nitrogen and hydrogen metabolism

To extend our understanding from the physiological studies shown in Figure 3, we monitored some key genes for carbon, nitrogen and hydrogen metabolism during phototrophic and chemotrophic growth. Compared to the photoheterotrophic growth of *H. modesticaldum*, in which energy is generated from light and reducing powers (NAD(P)H and Fd_red_) are generated from light and oxidation of organic carbon (i.e. pyruvate oxidation), less energy and reducing powers are expected to be generated for *H. modesticaldum *in darkness. In agreement with this hypothesis, most of the genes involved in energy metabolism are down-regulated during chemotrophic growth (Table 2 and Figure 4). For instance, genes encoding enzymes for (1) the EMP pathway (*pfkA *(HM1_0078), 6-phosphofructokinase, and *pykA *(HM1_0076), pyruvate kinase); (2) nitrogen assimilation (*nifD *(HM1_0864) and *nifK *(HM1_0865), subunits of molybdenum-dependent group I type (FeMo-co) nitrogenase); and (3) hydrogen production (*hupS *(HM1_1478) and *hupL *(HM1_1479), subunits of uptake nickel-iron ([NiFe])-hydrogenase (*hupSL*); *nuoEFG *(HM1_1028 - HM1_1029), encoding [FeFe]-hydrogenase; and *hymD *(HM1_1590), encoding the Hymd subunit of Fe-only dehydrogenase).

### (C) Pyruvate metabolism is either active or up-regulated in darkness

As shown in Figure 4, the expression level of genes presumed to carry out pyruvate metabolism during chemotrophic growth is either up-regulated, such as *porA *(HM1_0807, encoding PFOR; 4-8 fold increase), or not affected, as in the case for *fdxR *(HM1_0289, encoding ferredoxin (Fd)-NADP^+ ^oxidoreductase (FNR)) and two adjacent ferredoxin genes, *fdx *(HM1_1461) and *pshB *(HM1_1462). Despite the lack of genes encoding pyruvate dehydrogenase, PFOR can be an alternative enzyme for converting pyruvate into acetyl-CoA and Fd_red _in pyruvate fermentation (equation 1), and Fd_red _can interact with FNR, known to be the last electron transporter in the light-induced electron transfer chain, to produce NADPH (equation 2).(2)

Note that high FNR activity (10 μmole/min•mg protein) is detected in the cell free extract of *H. modesticaldum *(Additional file 5: Figure S4). Consistent with the studies of FNR from other organisms, we also detected that FNR in *H. modesticaldum *has higher specificity for NADPH versus NADH, and that the reaction turnover for producing Fd_red_, by measuring the formation of NADP^+ ^or NAD^+ ^(equation 2), is more than 50-fold faster for NADPH than for NADH (Additional file 5: Figure S4A). The rate of NADPH oxidation is accelerated with addition of ferricyanide (Additional file 5: Figure S4B). Together, the discovery of FNR activity in cell extracts indicates that the reducing power required for carbon and nitrogen metabolisms in *H. modesticaldum *can be generated from FNR during phototrophic and chemotrophic growth.

### (D) Photosynthetic pigments produced in darkness

**Figure 6:**
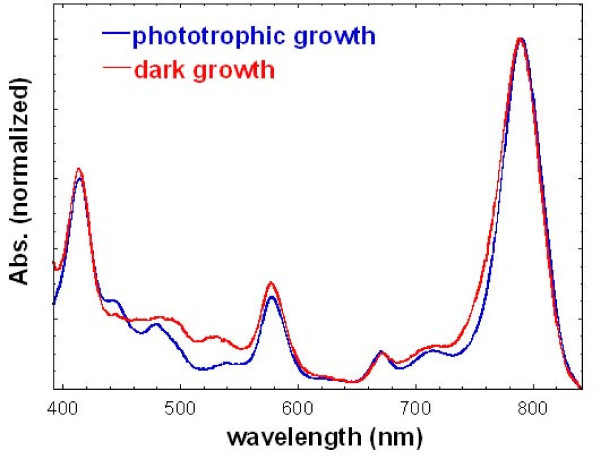
**Normalized absorption spectra of whole cell cultures during phototrophic and chemotrophic growth**. The cell scattering was digitally subtracted in the spectra.

The genomic information indicates that *H. modesticaldum *has the simplest (bacterio)chlorophyll biosynthesis pathway compared to other sequenced photosynthetic bacteria. A putative mechanism of BChl *g *biosynthesis was recently proposed [1]. The biosynthesis of photosynthetic pigments during chemotrophic growth under nitrogen fixing conditions has been observed for some species of heliobacteria, including *Heliobacillus mobilis*, *Heliobacterium gestii *and *Heliobacterium chlorum *[21]. Here, we would like to examine if *H. modesticaldum *can also produce (B)Chls in darkness. Figure 6 shows the normalized absorption spectra of the intact cell cultures from phototrophic and chemotrophic growth, after cell light-scattering has been digitally subtracted from the raw data (see Methods). The absorption peaks of the unique pigment BChl *g *at 788 nm and of 8^1^-OH-Chl *a*_F _at 670 nm can be detected in Figure 6, indicating that photosynthetic pigments can be produced by *H. modesticaldum *during chemotrophic growth. The expression levels of genes responsible for (B)Chls biosynthesis, *bchY *(chlorophyll reductase, subunit Y), *bchB *(protochlorophyllide reductase, subunit B), *bchE *(anaerobic cyclase) and *bchG *(bacteriochlorophyll synthase), are 2-16 fold lower in darkness than grown phototrophically (Figure 4 and Table 2).

### (E) Nitrogen is assimilated during phototrophic and chemotrophic growth

Biological nitrogen assimilation (i.e. diazotrophic growth) is an ancient process that is widely distributed in prokaryotes, and is found in some members of all groups of phototrophic bacteria [23]. Previous studies showed that nitrogen assimilation in heliobacterial cultures is "switched-off" when NH_4_^+ ^is supplied as the nitrogen source and activated with N_2_(g) supplied [6,24], and that *H. modesticaldum *is one of the only two known anaerobic anoxygenic phototrophs that can fix nitrogen at temperatures above 50°C [6,7]. Significant amounts of chemical energy (16 ATP) and reducing power (8 Fd_red_) are required during diazotrophic growth (N_2 _+ 8 H^+ ^+ 8 Fd_red _+ 16 ATP → 2 NH_3 _+ H_2 _+ 8 Fd_ox _+ 16 ADP + 16 P_i_) [25].

**Figure 7:**
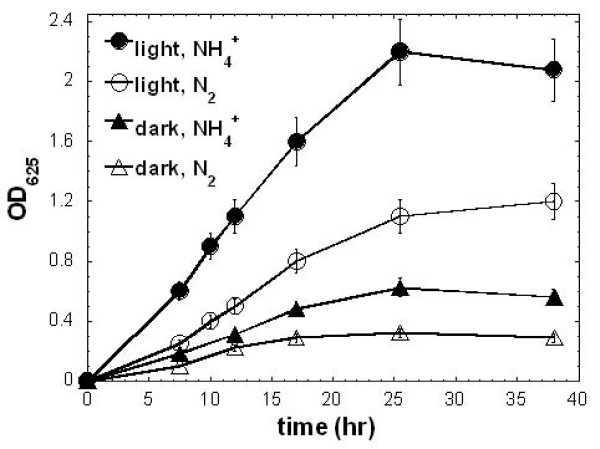
**Cell growth with or without nitrogen fixation in pyruvate-grown cultures during phototrophic and chemotrophic growth**. The cells were grown in the minimal medium with pyruvate as sole carbon source and NH_4_^+ ^or N_2_/H_2 _= 98/2 as the nitrogen source.

In the energy metabolism of *H. modesticaldum*, ATP and reducing power are required for carbon metabolism, nitrogen assimilation and hydrogen production. Because of the energy and reducing power demanded for nitrogen fixation, diazotrophic growth of *H. modesticaldum *in darkness may be very challenging. Figure 7 shows diazotrophic and non-diazotrophic growth during phototrophic and chemotrophic growth, and growth of *H. modesticaldum *is slower during diazotrophic growth. Table 3 indicates that a similar amount of acetate is excreted during diazotrophic and non-diazotrophic growth. Together, our studies suggest that *H. modesticaldum *generates sufficient chemical energy and reducing power for both carbon metabolism and nitrogen assimilation during chemotrophic growth.

## Discussion

### D-sugars are photoassimilated by *H. modesticaldum*

While the EMP pathway is annotated in the genome, no sugar-supported growth has been reported for *H. modesticaldum*. It is not uncommon for microorganisms that have the EMP pathway annotated but do not use glucose and other sugars as carbon sources, and to date only one heliobacterium, *Heliobacterium gestii*, has been reported to grow on C6-sugars, i.e. glucose and fructose [2]. Alternatively, fermentation of glucose through the EMP pathway has been reported in non-phototrophic bacteria in the phylum *Firmicutes *[26,27]. In this paper, we present the first report on the growth of *H. modesticaldum *supported by D-ribose, D-glucose and D-fructose with "vitamin-level" (0.02%) yeast extract included. With the support of multiple lines of experimental evidence, including physiological studies, activity assays, gene expression data, and mass spectra, our work addresses the puzzle that the EMP pathway has previously been annotated but no sugar-supported growth has been reported for *H. modesticaldum*.

The growth on D-ribose confirms the proposed function of a putative ribose ABC transporter (*rbsDACB*, HM1_2417 - HM1_2420) and ribokinase (*rbsK*, HM1_2416) through genome annotation, and growth supported by D-ribose, D-glucose and D-fructose suggests that annotated EMP and non-oxidative pentose phosphate pathways in *H. modesticaldum *are active in carbohydrate metabolism. As D-fructose and D-glucose are polar molecules, glucose, fructose or hexose transporter proteins are required to move those molecules across the cell membrane into the cells. No known hexose transporter has been reported for *H. modesticaldum*, which may partially explain slower growth on D-hexose than on D-ribose. It remains to be determined if the putative ribose transporter of *H. modesticaldum *functions as a hexose transporter, since no ribose transporter has been reported previously to accommodate a hexose.

Metabolism of carbohydrate through the EMP pathway supplies 2 ATP and 2 NADH to the cells, which are significant for the energy metabolism of *H. modesticaldum*, because essential genes in the oxidative pentose phosphate and ED pathways, which provide reducing equivalents during carbohydrate metabolism, are absent in the genome. Moreover, utilization of glucose can provide an additional path for H_2 _production in *H. modesticaldum *as reported in some non-phototrophic bacteria [28].

### The biological significance of the alternative CO_2_-assimilation pathways

The CO_2_-anaplerotic pathways are known to replenish the intermediates of TCA cycle, so that removal of the intermediates for synthesizing cell materials will not significantly slow down the metabolic flux through the TCA cycle. Our recent studies showed that the photoheterotrophic bacterium *R. denitrificans *uses the anaplerotic pathways to assimilate CO_2_. All of the genes encoding the enzymes for CO_2_-anaplerotic pathways, PEP carboxylase, PEP carboxykinase, pyruvate carboxylase and malic enzyme, have been annotated in the *R. denitrificans *genome, and activities of these enzymes have been detected. The alternative CO_2_-fixation pathways account for making up 10-15% cellular proteins of *R. denitrificans *[9]. Our studies presented here also suggest that *H. modesticaldum *uses two anaplerotic pathway, PEP carboxykinase (PEPCK) and pyruvate:ferredoxin oxidoreductase (PFOR), for assimilating CO_2_.

What is the biological importance of PEPCK and PFOR in *H. modesticaldum*? Although the anaplerotic CO_2_-assimilation cannot support (photo)heterotrophic growth in the way that the autotrophic CO_2_-fixation supports (photo)autotrophs, these two CO_2_-anaplerotic pathways are critical for the carbon metabolism of *H. modesticaldum *(see Figure 5). First, the CO_2_-assimilation by PFOR catalyzes the formation of pyruvate from acetyl-CoA, a reaction that cannot be catalyzed by pyruvate dehydrogenase. Hence, PFOR is essential for acetate assimilation and pyruvate metabolism. Second, as shown in Figure 5, PEPCK is required to convert PEP into OAA in the partial reductive TCA (rTCA) cycle. Without assimilating CO_2 _by PEPCK, carbon flux through the partial rTCA cycle cannot take place.

### Possible functions of PFOR and FNR during chemotrophic growth

**Figure 8:**
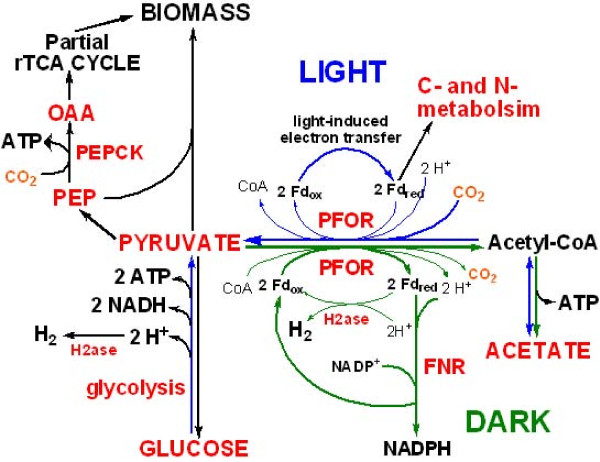
**Summary of energy metabolism of *H. modesticaldum *during phototrophic and chemotrophic growth described in this report**. Bold curves and lines represent the proposed major pathways during phototrophic (shown in blue) and chemotrophic (shown in green) growth. Abbreviation: Fd_ox _or Fd_red_, the oxidized or reduced form of ferredoxin; FNR, ferredoxin-NADP^+ ^oxidoreductase; H2ase, hydrogenase; OAA, oxaloacetate; PFOR, pyruvate:ferredoxin oxidoreductase; PEP, phosphoenolpyruvate; PEPCK, PEP carboxykinase; rTCA, reductive TCA cycle.

To evaluate the function of PFOR and FNR in pyruvate metabolism in darkness, we examine the culture growth in acetate-supported medium with and without the addition of HCO_3_^- ^and acetate excretion from pyruvate-grown cultures. No CO_2_-enhanced growth in acetate-supported medium can be detected, and cell growth in acetate medium is extremely slow in darkness (data not shown). Also, approximately 44% of the pyruvate in pyruvate-grown cultures is converted into acetate during chemotrophic growth (Table 3). Madigan and coworkers reported a large amount of CO_2 _by analyzing the gas phase of chemotrophic-grown heliobacterial cultures [21]. Together, the following roles of PFOR and FNR during chemotrophic growth can be proposed (Figure 8): (1) *PFOR provides energy and reducing power for cellular functions*. PFOR catalyzes pyruvate fermentation to acetyl-CoA, CO_2_, 2 Fd_red _and 2 H^+ ^(equation 1). Fd_red _is used for carbon and nitrogen metabolism in darkness (Figure 7), and 2 Fd_red _and 2 H^+ ^from the oxidation of pyruvate can generate H_2 _by [FeFe]-hydrogenase (2 Fd_red _+ 2 H^+ ^→ 2 Fd_ox _+ H_2_) (Figure 8). 2 Fd_ox _can be then used for pyruvate fermentation. Further, acetyl-CoA can be utilized to generate acetate and produce ATP through substrate-level phosphorylation catalyzed by ACK (Table 3 and Figure 5). This ATP production process may partially explain pyruvate being the most favorable nutrition source; and (2) *FNR produces NADPH during chemotrophic growth*. As mentioned above, essential genes in the oxidative pentose phosphate and ED pathways, two potential sources producing NADPH, are missing in the *H. modesticaldum *genome. While NADPH is generated by FNR via the light-induced electron transfer during phototrophic growth, NADPH production is also required during chemotrophic growth. It is likely that some Fd_red _molecules produced by pyruvate fermentation in *H. modesticaldum *are used to produce NADPH by FNR during chemotrophic growth (equation 2). When this occurs, Fd_ox _is regenerated for pyruvate fermentation (Figure 8). In summary, since [FeFe]-hydrogenase and FNR compete for using 2 Fd_red _and 2 H^+ ^produced from pyruvate fermentation, intracellular NAD(P)H availability likely plays important role on H_2 _production, as well as nitrogen and carbon flux, in *H. modesticaldum*.

## Conclusions

*H. modesticaldum *is one of the only two cultured anoxygenic phototrophs that can fix nitrogen at temperatures above 50°C. Only acetate, lactate and pyruvate have been reported previously to support the photoheterotrophic growth of *H. modesticaldum*, and it is necessary to further explore carbon sources in order to understand energy metabolism in-depth. In this paper, we developed the growth medium close to a minimal growth medium, and report the first studies, with comprehensive experimental evidence supported, that D-ribose, D-glucose and D-fructose can be photoassimilated as sole carbon sources to generate cell material. Additionally, in the absence of autotrophic CO_2 _fixation, *H. modesticaldum *uses two CO_2_-anaplerotic pathways during phototrophic growth: pyruvate:ferredoxin oxidoreductase (PFOR) and phosphoenol-pyruvate carboxykinase (PEPCK). The CO_2_-anaplerotic pathways by PFOR and PEPCK are essential for acetate assimilation, pyruvate metabolism and introducing carbon flow into the rTCA cycle for generating cell materials, including photosynthetic pigments (Figure 5).

Our studies suggest that PFOR and ferredoxin-NADP^+ ^oxidoreductase (FNR) are required for generating reducing power (Fd_red _and NAD(P)H) during chemotrophic growth. A similar ratio of acetate excretion/pyruvate consumption is observed in pyruvate-grown cultures during phototrophic versus chemotrophic growth, and conversion of acetyl-CoA to acetate can generate ATP for the energy required for *H. modesticaldum *in darkness. Also, since energy and reducing power produced by *H. modesticaldum *during chemotrophic growth are rather limited compared to phototrophic growth, cellular functions demanding high-energy input, such as nitrogen fixation and hydrogen production, are down-regulated. Nevertheless, our studies indicate that *H. modesticaldum *produces sufficient energy and reducing power for both carbon metabolism and nitrogen fixation during chemotrophic growth, albeit at a relatively low growth rate. An overview of energy metabolism pathways of *H. modesticaldum *is shown in Figure 8. In summary, our reported studies not only significantly broaden our current knowledge, but also provide new and essential insights on the energy metabolism of *H. modesticaldum*.

## Methods

### Materials

Chemicals and enzymes for enzymatic activity assays were purchased from Sigma-Aldrich. The ^13^C-labeled glucose and pyruvate were from Cambridge Isotope Laboratories (CIL), Inc. The DNA oligomers were from Integrated DNA Technology (IDT) without further purification. The source culture of *Heliobacterium modesticaldum *Ice1^T ^was a gift from the laboratory of Dr. Michael T. Madigan at Southern Illinois University, Carbondale.

### The growth of bacterial strains

All *Heliobacterium modesticaldum *Ice1^T ^cultures reported in this work were grown anaerobically inside a Coy anaerobic chamber at temperatures ranging from 46-50°C, and cell growth was estimated turbidimetrically at OD_625_. OD_625 _was chosen for evaluating the cell growth because absorbance of photosynthetic pigments is minimal around 625 nm (as shown in Figure 6). Phototrophic cultures were grown in low-intensity light (10 ± 1 W/m^2^), and chemotrophic cultures were grown in darkness. The list of growth media used in this report and organic carbon sources included in each medium are described in Table 1. The pyruvate mineral salts (PMS, with 20 mM (2.2 g/L) pyruvate included) medium were prepared as reported previously [2]. The chemicals in yeast extract (YE) medium (per liter) are: K_2_HPO_4 _(1.0 g), MgSO_4_•7H_2_O (0.2 g), CaCl_2_•2H_2_O (20 mg), Na_2_S_2_O_3_•5H_2_O (0.2 g), yeast extract (4.0 g), (NH_4_)_2_SO_4 _(1.0 g), chelated iron solution [21] (2 ml), *d*-biotin (15 μg), vitamin B_12 _(20 μg) and trace element solution (1 ml) with the final pH adjusted to pH 6.9-7.0. Components of the trace element solution were reported previously [2]. Pyruvate (20 mM for phototrophic growth and 40 mM for chemotrophic growth) is added to YE medium to prepare pyruvate-yeast extract (PYE) medium. Sodium acetate (40 mM) and HCO_3_^- ^(20 mM) are included in acetate-mineral salts (AMS) medium, and sugar (hexose or ribose) (40 mM) and "vitamin level" yeast extract (0.02%) are included in sugar-grown medium. Cultures of *H. modesticaldum *were grown either photoheterotrophically in PMS, YE, PYE, AMS and different sugar-grown medium (listed in Table 1) or chemotrophically (dark, anoxic) in PYE medium. NH_4_Cl (in mineral salts medium), (NH_4_)_2_SO_4 _(in YE and PYE medium), and N_2_/H_2 _= 98/2 (under nitrogen fixation conditions) was used as the nitrogen source. Typically, 1-2% cultures (50-100 fold dilution) in the late exponential growth phase were used to inoculate fresh media.

### Measurement of the uptake of pyruvate, acetate, lactate, fructose and glucose

The amount of pyruvate and lactate in the cultures of *H. modesticaldum *under different growth conditions was determined by the methods reported previously [9,29]. The amount of D-glucose and pyruvate in the cultures of *H. modesticaldum *under different growth conditions was determined by the methods reported previously [9]. Uptake of D-fructose was estimated by a coupled hexokinase/phosphoglucose isomerase/glucose-6-phosphate dehydrogenase assay, and the amount of NADPH formed in the reaction, measured by the increase of the absorbance at 340 nm, is stoichiometric to the amount of D-fructose in solution. The amount of acetate production was determined by a coupled acetyl-CoA synthase/citrate synthase/malate dehydrogenase assay following the formation of NADH [30].

### RNA extraction and quantitative real-time PCR (QRT-PCR)

The methods used to extract RNA and perform QRT-PCR were described previously [9,31]. QRT-PCR was performed to profile the gene expression under different growth conditions of *H. modesticaldum*. The primers for QRT-PCR in this report are listed in Additional file 6: Table S2. Two biological replicates, with three technical replicates for each biological sample, were performed for validation, and the mean value was reported. The amplified DNA fragments were verified by 1% agarose gel electrophoresis, and a single fragment was obtained for all amplicons.

### Mass spectrometry

Photosynthetic pigments in *H. modesticaldum *cultures were extracted as reported previously [10]. The mass spectra of the photosynthetic pigments, BChl *g *and 8^1^-OH-Chl *a*_F_, were acquired using matrix-assisted laser desorption ionization-time-of-flight (MALDI-TOF) mass spectrometry. Sample measurements and preparation were described previously [31].

### Cell scattering subtraction of the absorption spectrum

The light scattering of cells was digitally subtracted from the raw data of Figure 6 using the approach described as follows. The scattering presented in the raw data of the original spectrum was first mimicked by an analytical function, , in which a, b, c are variable coefficients and λ is wavelength (nm). An initial function was applied in the long wavelength range, where the pigments absorption does not contribute to the scattering. The fitting equation has been written and applied in Origin 7.5 (Origin Lab Corp.). After obtaining the formula, a scattering simulation was extrapolated to the short wavelength range and subtracted from the original spectrum.

### Activity assays

Enzymatic activities were performed with cell-free crude extracts prepared as follows: Cells were harvested by centrifugation at 5,000 × *g *for 15 min at 4°C and washed with 20 mM Tris-HCl buffer at pH 8.0. The cell pellet was resuspended in the same buffer containing 1 mM phenylmethanesulfonyl fluoride (PMSF). Resuspended cells were disrupted by sonication, and cell debris was removed with centrifugation at 20,000 × *g *for 30 min. Protein concentration in cell extracts was determined by the Bradford assay [32] using bovine serum albumin as a standard. The enzymatic activity of acetyl-CoA synthetase, acetate kinase, ATP citrate lyase, citrate synthase, ferredoxin-NADP^+ ^oxidoreductase, hexokinase, phosphenolpyruvate carboxykinase, 6-phosphofructokinase, pyruvate kinase and phosphotransacetylase in cell-free extracts was assayed as described previously [16,18,33-39].

## Authors' contributions

KHT performed the cultivation experiments, gene expression assays, and activity measurements, carried out the labeling studies, participated in the design of the study, and drafted the manuscript. HY performed the cultivation experiments and gene expression assays together with KHT. REB conceived, designed and coordinated the study. All authors read and approved the final manuscript.

## Supplementary Material

Additional file 1 **Figure S1: Effect of glucose and fructose on cell growth of *H. modesticaldum***. Growth curve in YE growth medium (described in Methods and Table 1) supplied with 40 mM fructose, 40 mM glucose, or no defined organic carbon included (panel A) and with different concentration of glucose (0, 1.25, 2.5, 5, 10, 20 and 40 mM) (panel B). Click here for file

Additional file 2 Figure S2: Growth of *H. modesticaldum *on pyruvate and acetate in the presence of yeast extract. Click here for file

Additional file 3 Table S1: Expression levels of genes in cultures of PYE and PMS growth media. Click here for file

Additional file 4 **Figure S3: Activity assay of ATP citrate lyase (ACL) in the cell extracts of *Cba. tepidum *and *H. modesticaldum***. The assays were performed as described previously [16] (see ref. [16]). The formation of oxaloacetate catalyzed by ACL was coupled to the oxidation of NADH oxidation, probed by the decrease at A_340_, catalyzed by malate dehydrogenase. Click here for file

Additional file 5 **Figure S4: Activity assay for ferredoxin-NADP^+ ^oxidoreductase **(**FNR) in the cell extracts of *H. modesticaldum***. The reaction turnover is monitored by the oxidation of NADPH or NADH at the decrease of A_340 _with the procedure reported previously [34] (see ref. [34]). **Activity assay of NADH versus NADPH **(panel A): 0.25 mM NADH or NADPH was used for assaying activity. The reaction rate with NADPH is > 50 fold faster than with NADH, and **effect of ferricyanide in the activity of FNR with NADPH as the substrate **(panel B). Click here for file

Additional file 6 Table S2: Sequences of primers used for QRT-PCR studies reported in this paper. Click here for file
