# Supplementary figures and images for: Energy metabolism of Heliobacterium modesticaldum during phototrophic and chemotrophic growth
Source: BMC Microbiol. 2010 May 24;10:150. doi: 10.1186/1471-2180-10-150 (PMC2887804; doi:10.1186/1471-2180-10-150)

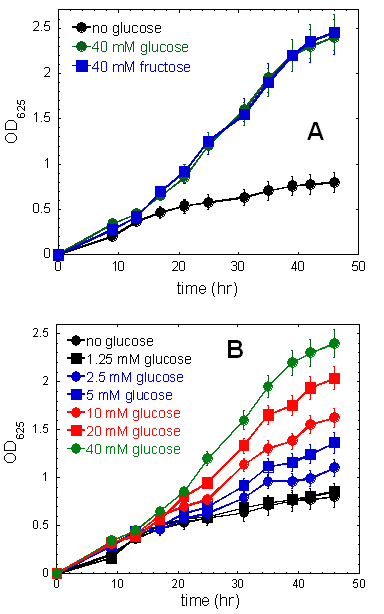

Supplement: Additional file 1 — Figure S1: Effect of glucose and fructose on cell growth of H. modesticaldum. Growth curve in YE growth medium (described in Methods and Table 1) supplied with 40 mM fructose, 40 mM glucose, or no defined organic carbon included (panel A) and with different concentration of glucose (0, 1.25, 2.5, 5, 10, 20 and 40 mM) (panel B). [file 1471-2180-10-150-S1.BMP]

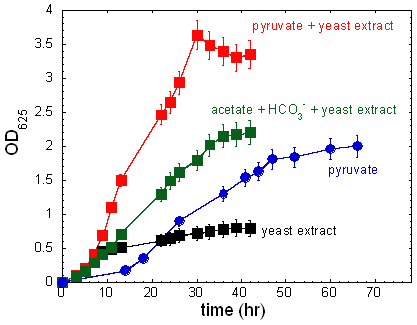

Supplement: Additional file 2 — Figure S2: Growth of H. modesticaldum on pyruvate and acetate in the presence of yeast extract. [file 1471-2180-10-150-S2.BMP]

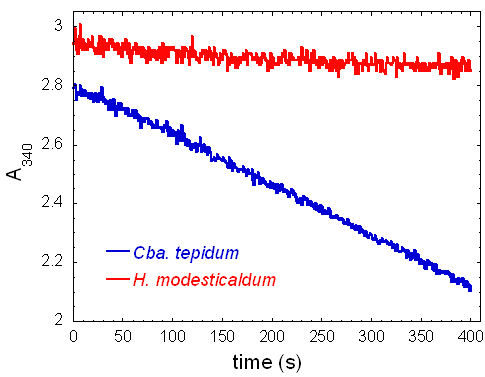

Supplement: Additional file 4 — Figure S3: Activity assay of ATP citrate lyase (ACL) in the cell extracts of Cba. tepidum and H. modesticaldum. The assays were performed as described previously [16] (see ref. [16]). The formation of oxaloacetate catalyzed by ACL was coupled to the oxidation of NADH oxidation, probed by the decrease at A340, catalyzed by malate dehydrogenase. [file 1471-2180-10-150-S4.BMP]

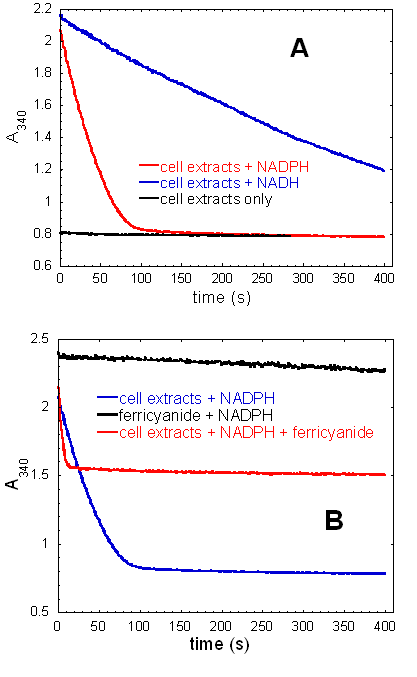

Supplement: Additional file 5 — Figure S4: Activity assay for ferredoxin-NADP+ oxidoreductase (FNR) in the cell extracts of H. modesticaldum. The reaction turnover is monitored by the oxidation of NADPH or NADH at the decrease of A340 with the procedure reported previously [34] (see ref. [34]). Activity assay of NADH versus NADPH (panel A): 0.25 mM NADH or NADPH was used for assaying activity. The reaction rate with NADPH is > 50 fold faster than with NADH, and effect of ferricyanide in the activity of FNR with NADPH as the substrate (panel B). [file 1471-2180-10-150-S5.BMP]
