# Supplementary material for: Energy metabolism of Heliobacterium modesticaldum during phototrophic and chemotrophic growth
Source: BMC Microbiol. 2010 May 24;10:150. doi: 10.1186/1471-2180-10-150 (PMC2887804; doi:10.1186/1471-2180-10-150)
Supplement: Additional file 3 — Table S1: Expression levels of genes in cultures of PYE and PMS growth media. [file 1471-2180-10-150-S3.DOC]

**Table S1. Expression levels of genes in cultures of PYE and PMS growth media**

| **Gene** | **ΔC_T_ in PYE culture** | **ΔC_T_ in PMS culture** | **ΔΔC_T_ (ΔC_T_ in PYE-ΔC_T_ in PMS)** | **Relative gene expression level (PYE/PMS; = 2^-ΔΔC^_T_)** |
| --- | --- | --- | --- | --- |
| *pfkA* (6-phosphofructokinase) | 15.0 ± 0.1 | 16.2 ± 0.1 | -1.2 | 2.3 |
| *pykA* (pyruvate kinase) | 13.5 ± 0.1 | 13.9 ± 0.1 | -0.4 | 1.3 |
| *porA* (pyruvate:Fd oxidoreductase) | 13.7 ± 0.1 | 13.5 ± 0.1 | 0.2 | 0.9 |
| *fdxR* (Fd-NADP^+^ reductase, FNR) | 14.7 ± 0.1 | 14.5 ± 0.1 | -0.2 | 1 |
| ferredoxin (for FNR) | 13.4 ± 0.1 | 13.5 ± 0.1 | -0.1 | 1.1 |
| *pshB* (PshB in RC) | 14.0 ± 0.1 | 14.0 ± 0.0 | 0 | 1.0 |
| *ackA* (acetate kinase) | 10.6 ± 0.1 | 9.9 ± 0.2 | 0.7 | 0.6 |
| *acsA* (acetyl-CoA synthase) | 15.5 ± 0.1 | 16.1 ± 0.1 | -0.6 | 1.5 |
| *ppdK* (pyruvate phosphate dikinase) | 13.4 ± 0.1 | 14.7 ± 0.2 | -1.3 | 2.5 |
| *pckA* (PEP carboxykinase) | 14.1 ± 0.1 | 14.7 ± 0.1 | -0.6 | 1.5 |
| *mdh* (malate dehydrogenase) | 14.5 ± 0.1 | 14.9 ± 0.1 | -0.4 | 1.3 |
| *bchY* | 13.1 ± 0.1 | 11.5 ± 0.0 | 1.6 | 0.3 |
| *bchB* | 14.2 ± 0.1 | 13.0 ± 0.0 | 1.2 | 0.4 |
| *bchE* | 13.2 ± 0.1 | 12.1 ± 0.0 | 1.1 | 0.5 |
| *bchG* | 12.9 ± 0.1 | 11.8 ± 0.1 | 1.1 | 0.5 |
| *nifK* ([FeMo]-nitrogenase, β subunit) | 13.0 ± 0.0 | 11.7 ± 0.0 | 1.3 | 0.4 |
| *nifD* ([FeMo]-nitrogenase, α subunit) | 13.7 ± 0.0 | 11.6 ± 0.1 | 2.1 | 0.2 |
| *hupS* ([NiFe]-hydrogenase, small subunit) | 13.3 ± 0.1 | 10.8 ± 0.1 | 2.5 | 0.2 |
| *hupL* ([NiFe]-hydrogenase, large subunit) | 12.7 ± 0.1 | 10.1 ± 0.1 | 2.6 | 0.2 |
| *hymD* (Fe only hydrogenase, hymd subunit) | 13.4 ± 0.1 | 11.5 ± 0.0 | 1.9 | 0.3 |
