# Supplementary material for: Energy metabolism of Heliobacterium modesticaldum during phototrophic and chemotrophic growth
Source: BMC Microbiol. 2010 May 24;10:150. doi: 10.1186/1471-2180-10-150 (PMC2887804; doi:10.1186/1471-2180-10-150)
Supplement: Additional file 6 — Table S2: Sequences of primers used for QRT-PCR studies reported in this paper. [file 1471-2180-10-150-S6.DOC]

**Table S2. Sequences of primers used for QRT-PCR studies reported in this paper.**

| Gene (loci number, predicted function) | Forward primers (5’🡪 3’) | Reverse primers (5’🡪 3’) |
| --- | --- | --- |
| **Genes for carbon metabolism** | | |
| *pykA* (HM1_0076, pyruvate kinase) | GCCCGAATCATCTCCATCAG | AACGCCCCGCACGAA |
| *PfkA* (HM1_0078, 6-phosphofructokinase) | CCATCACCTCGATCACATTCG | GTCCTCGACGCTATCAACAAGA |
| *fdxR* (HM1_0289, ferredoxin-NADP^+^ reductase, FNR) | CCTGCTCCCGGTCAAAATC | TTCTTCGCGCCGATGAA |
| *porA* (HM1_0807, pyruvate:ferredoxin oxidoreductase, PFOR) | GAAGCCTGCAACCCCTACTATAAG | GGTGAGTTTGCCGATCTCCTT |
| *acsA* (HM1_0951, acetyl-CoA synthetase) | TCCAAACCTGAAATCCTATGAAGAG | AGAACTCGCGCTCCACATCT |
| HM1_1461, ferredoxin | CCCGTGAATGCAATCATCAA | TGGGCAAGTGTCAGCACAAG |
| *pshB* (HM1_1462, RC polypeptide, PshA) | GGCTTACAAAATCACCGATGCT | GCACTTGTCGGCGCAAAC |
| *mdh* (HM1_1472, malate dehydrogenase | CGGCTATGAGGGCATCTACAC | CGGTCAGCTCGATCTCAAAGA |
| *pykA* (HM1_1600, pyruvate kinase) | GGCGGTAAGGTCTCCAACAA | ACCGAGGGCAGGTTGATG |
| *ackA* (HM1_2157, acetate kinase) | CCCGCGTCGGTGACAT | CGTCAATCCCTCTTTTTCCATC |
| *ppdK* (HM1_2461, pyruvate phosphate dikinase) | AGATGTCGTTGCCGGTATCC | AAGCATTCGGGCAGTTCTTC |
| *pckA* (HM1_2773, phosphoenolpyruvate carboxykinase) | GATGCCATCTTCCACGAGGTA | CAGTCCCTGTTACGTGTCGAAA |
| **Genes for (bacterio)chlorophyll biosynthesis** | | |
| *bchY* (HM1_0654, chlorophyll reductase, subunit Y) | GAAGTGGGCGAGGAAATTGA | CTGGAAGGTTTTCTCCCTATTCTG |
| *bchB* (HM1_0685, protochlorophyllide reductase, subunit B) | GATCGTGCCCCAGGTGAAC | TGGGCCAGAAAGTCATCCAT |
| *bchE* (HM1_0688, anaerobic cyclase) | CCGAAGCGGCCACTCA | GTTCTCTTCCATCGTCGTTTCC |
| *bchG* (HM1_0692, bacteriochlorophyll synthase) | ACGTGACCCTGCCTTGGA | TCGTCTGTTCCGGCGTTAC |
| **Genes for N_2_ fixation and H_2_ production** | | |
| *nifK* (HM1_0864, nitrogenase Fe/Mo protein, β subunit) | CACCGTCGGCAGCTACAAGT | TGATACCGATGGGCATGGT |
| *nifD* (HM1_0865, nitrogenase Fe/Mo protein, α subunit) | CGGCCCCACTAAGATCAAAG | TGGATGGTCTCGTCGAAGAAA |
| *hupS* (HM1_1478, [NiFe]-hydrogenase small subunit) | CTGCCGCCAATCCGAAT | ATGGGCTTGTCTTTGATGATCTC |
| *hupL* (HM1_1479,  [NiFe]-hydrogenase large subunit) | CCGAACTGATCCGCAACAT | GGCGTGCAGATGGTAGAAGTG |
| *hymD* (HM1_1590,  Fe only hydrogenase, hymd subunit) | GCGGCGGCAGTGAATTAT | CAACGAGAGCGCCTTTGC |
| *nouE* (fused gene, HM1_1028) | CAGATCGGACAAAAAGGGAAAC | TTCCCGATATTTTTCCAGCAA |
| *nuoF* (fused gene, HM1_1028) | AGTTACCCGGAGATTCCCTTCT | GGTTGATGTGACCGCAGTTG |
| *nuoG* (HM1_1029) | GGCGCGATGATCGTATCAA | AGAGCATCTGCGCCAAGGT |
